# Decision-making for birth location among women in Pakistan: evidence from national survey

**DOI:** 10.1186/s12884-018-1844-8

**Published:** 2018-06-14

**Authors:** Muhammad Iftikhar ul Husnain, Mudassar Rashid, Usman Shakoor

**Affiliations:** COMSATS University, Islamabad, Pakistan

**Keywords:** Home birth, Logistic regression, Women’s empowerment, Pregnancy, Pakistan

## Abstract

**Background:**

Pakistan ranks 149th in the maternal mortality ratio (MMR) and has failed to keep pace with other countries in the region, except Afghanistan, with respect to health indicators. Home deliveries are linked to a higher risk of maternal death; therefore, discouraging home deliveries is imperative to improve maternal health. This study provides a holistic view and analyses factors affecting home birth decisions within the context of maternal socio-demographic characteristics in Pakistan.

**Methods:**

The study exploits the latest data from the Pakistan Demographic and Health Survey (2012–2013), which includes a nationally representative sample of 13,558 women aged 15–49 years. However, the sample was reduced to 6977 women who had given birth in the 5 years preceding the survey. Statistical techniques, including bi-variate and multivariate logistic regression, were used to analyse the data. The dependent variable was dichotomous and coded as 0 for home deliveries and 1 for deliveries at a health facility. The dependent variable was constructed based on information regarding the most recent birth in the 5 years preceding the survey.

**Results:**

The study reveals that giving birth at home is highly prevalent among mothers in Pakistan (Baluchistan, 74%; Khyber-Pakhtunkhwa, 53%; Gilgit Baltistan, 46%; Punjab, 45% and Sindh, 34%) because of their difficulty obtaining permission to visit a health facility, financial barriers, the distance to health facilities and transportation. Substantial variation is observed when geo-demographic characteristics are considered. Higher home childbirth rates have been recorded in rural areas compared with those in urban areas (OR 1.32; *p* ≤ 0.000). The likelihood of home birth is highest (OR 2.67; *p* = 0.000) among women in Baluchistan province and lowest (OR 0.48; *p* = 0.000) among mothers in Punjab province. After controlling for all odds ratios and demographic characteristics, the parents’ education levels remain a significant factor (p = 0.000) that affects women’s decisions to deliver at home rather than at a health facility.

**Conclusion:**

The study findings provide a better understanding of why women prefer to give birth at home. These results can help policymakers to introduce appropriate interventions to increase the number of deliveries at health facilities. These findings are expected to reduce maternal and neonatal mortality in Pakistan.

## Background

Developing countries still face undesirable outcomes of the mother’s decisions to deliver at home, while developed countries are discussing a woman’s right to choose between home or hospital childbirth [1–4]. Nearly all maternal deaths can be prevented when mothers deliver at a health facility instead of at home [5] because this decision decreases the likelihood of death for both the baby and the mother [6]. Unfortunately, 536,000 women die every year due to pregnancy complications [7] because many women prefer to deliver at home without professional medical assistance in low-income settings [8].

Although maternal and child mortality has decreased significantly in the last few decades in Pakistan, this measure has not reached the target set by Millennium Development Goal (MDG) five (75% reduction in maternal mortality by 2015). According to the Pakistan Bureau of Statistics, every 20 min, a mother dies in Pakistan due to pregnancy-related complications because mothers still prefer to deliver at home. Women visit health facilities only for serious and fatal complications [9], and only a small proportion of women (23%) visit health care facilities [10]. Therefore, maternal morbidities go unnoticed or are mismanaged [11]. Roughly, 59 out of 1000 women die due to maternal causes in Pakistan [12].

Decision making in pregnancy and birth is challenging for women due to indirect nature of information that frames decisions and presumably comes from outside sources as health professionals [13]. Lack of skilled birth attendants affects decisions to deliver at home or health facility. Sri Lanka has reduced number of maternal mortalities significantly by producing vital healthcare skills in rural areas (http://www.who.int/workforcealliance/forum/2011/hrhawardscs28/en/). The availability of skilled birth attendant during childbirth decreases the probability of death of either mother or baby [6]. The majority of pregnancy related deaths and disabilities could be averted by ensuring mother’s access to skilled birth attendants [14]. It is a proven fact that utilization of institutional delivery service reducing number of maternal health [15].

The reason why most deliveries still take place at home has been an area of interest for researchers worldwide, and recently, many studies have tried to understand the determinants of home births [7, 8, 16–18]. To the best of our knowledge only one study, by Javed et al. [7], has investigated factors associated with home versus hospital deliveries in Pakistan and document that mothers having lower levels of education, living in rural areas, residing in provinces other than Punjab, working in agriculture sector and relatively young mothers are more likely to give births at home.

However, our work improves upon their study in at least five respects. First, we use the latest data from the Pakistan Demographic and Health Survey (PDHS) [19] conducted from 2012 to 13, which provides rich information on mother’s decisions to deliver at home or in a health facility compared to the previous survey conducted from 2006 to 07. Second, we significantly increased the sample size (35%), which was expected to enhance the reliability of the results obtained in this study. Third, this data set separately provides information on a newly created province named Gilgit Baltistan (GB), and this study is expected to provide knowledge about factors influencing home birth decision making among mothers in this province for the first time. Fourth, instead of comparing whether women discuss their pregnancy issues with their husbands, we adopt a broader approach by comparing whether the mother or someone else makes the decision concerning the place of birth. This variable is more informative in the context of Pakistani cultural and social norms. Finally, receiver operating characteristic (ROC) curve testing was used to analyse the predictive power of the estimated models, which has been frequently overlooked by previous empirical studies.

### Literature review

Only one prior study has analysed the determinants of the mother’s choice to deliver at home or in a health facility in Pakistan. That study reported that a high healthcare cost was the major factor that restricted women from delivering babies at health centres. The parental education levels are key to decision making, with the likelihood of delivering at home decreasing as the parents’ education levels increase [7]. Female empowerment also reduces the likelihood of delivering a baby at home. Rural women are more likely to have home births compared to urban mothers. Women working in the agricultural sector have an increased probability of delivering babies at home. Previous literature establishes that community beliefs and norms about health behaviours strongly influence the decisions made by individuals to seek care [20]. Due to strong cultural norms, the probability of home birth is higher in Baluchistan Province [8]. These norms include denial of women education as it brings no return to parents [21], consideration of women inferior to men both physically and mentally, early marriage of daughters, confinement of women at homes, forcing them to observe purdah (clad), not allowing girls to go alone elsewhere [22, 23], not inclusion of women in decision making, decision of women fate by family men [24] discrimination of health treatment to females [25] and non-acceptance of women share in heritage among others.

The empirical literature shows that education has the largest impact on institutional delivery, which is preferred by educated women. Transportation barriers, lower autonomy, long distances from a health facility, lower levels of household income and no exposure to media can substantially lower the use of institutions as birth places [6, 26]. Financial constraints and lack of awareness regarding the importance of maternal health services remain fundamental predictors for not delivering babies at health facilities in Pakistan [27]. Urban residence strongly determines the use of skilled birth attendants but does not necessarily lead to the use of antenatal care [28, 29]. Cash incentives positively influence women’s decisions, whereas poor geographical access increases the likelihood of home birth [20]. Joint decision-making with families, household dynamics and perceived quality of care are also influential in determining a mother’s decision to seek care [16]. The positive attitudes of health workers and understanding that complications during labour or delivery can be properly managed at a health facility also increase the probability that women will choose to give birth at a health institution [17]. The education level of the husband and distance less than five kilometres from a health facility are significantly linked with institutional birth service utilization [30]. A lack of privacy, short labour, the pattern of decision making power within the household, tradition and culture are key determinants of the childbirth location. The decision of birth location substantially varies among different ethnic groups, and young mothers are more likely to deliver at home than older mothers [18]. Traditional views, fear of a high risk of having a caesarean at a hospital, religious fallacy, and a dearth of female doctors at health facilities increase the likelihood of home birth [31]. A high economic status, history of abortion, and having two or fewer children are reported to be positively and substantially linked with a woman’s preferences to use antenatal care facilities in Pakistan. A study in Nepal found that home visits by outreach workers increased the utilization of health services [32], whereas several other studies concluded that quantification of home visiting programs was difficult [33]. A study from Pakistan concluded that home visits from female health workers increased the utilization of antenatal services (ANC) [34]. The impact of health insurance on ANC service use has been found to be equally important as household wealth, maternal education, and urban residence in Turkey [28]. It is proven fact that by providing training to women in rural communities about birth related issues can significantly decrease the number of deaths and disabilities during pregnancy (http://www.who.int/workforcealliance/forum/2011/hrhawardscs28/en/), [6, 14].

## Methods

The data set was extracted from the publicly available PDHS 2012–13, which provides information on many health indicators, including maternal health. The survey consisted of a sample of 13,538 ever-married women of reproductive age (15–49 years) who were selected using multistage clustering sampling from all over Pakistan. For this analysis, information was collected on participants whose last child was born in the 5 years preceding the survey (*N* = 6977); this information is available in the family planning, pregnancy, and postnatal section of the survey. Bivariate and multivariate logistic regression analyses were used for the data analysis to achieve the study objectives. The dependent variable distributions of the selected characteristics, frequencies and cross-tabulations were used in the bivariate analysis, whereas the multivariate logistic regression analysis was used to assess associations between the decision to birth and other variables. No obligation for ethical clearance was necessary, since the study involved secondary and publicly available data.

### Measures

The dependent variable was dichotomous and was coded as “0” if the baby was delivered at home and as “1” if the baby was delivered in a location other than the home. Government health centres and public and private hospitals were considered other health facilities. Table 1 describes how the variables were constructed.

**Table 1:** Variables description

S. No	Variable	Construction
1	Education of Women	No education*, Primary, Secondary, Higher
2	Education of Husband	No education*, Primary, Secondary, Higher
3	Women Occupation	Not working*,Agriculture, Sales, Others
4	Decision of woman’s medical treatment	Self*, Others
5	Age of women	15–24*,25–34,35–49
6	Regions	Punjab*, Sindh, KPK, Baluchistan, Gilgit Bultistan
7	Residence	Urban*, Rural
8	Economic Status	Rich*, Poor, Middle
9	Transport Availability	Problem*,Not a Problem
10	Travelling Alone	Problem*,Not a Problem

*Reference Category

All independent variables used in the analysis were chosen based on prior empirical literature on the determinants of the mother’s decisions to deliver at home. The mother’s education was categorised as no education or primary, secondary, or higher education. The education of the husband is equally important in this scenario; therefore, this variable was constructed as described for the mother’s education. The economic status of women has been a key factor in the decision to deliver at home or in a hospital/clinic. This variable is captured through the following categories: poor, middle and rich. Women’s occupations are also important in our context; therefore, the following groups were created to capture this variable: not working, agriculture, sales and other work. The rural-urban division of the sample helped elucidate cultural and infrastructural differences. Rather than using whether women discussed their pregnancy issues with their husbands or not as a variable, we adopted a broader category concerning whether the mother or someone else made the decision concerning the place of birth. This variable is more informative in the context of Pakistani cultural and social norms. Three age groups (15–24, 25–34 and 35–49 years) were purposefully framed to measure the current ages of the women. The geographical division of the sample was represented by five provinces, and data from the newly created province Gilgit Baltistan were collected separately in a Pakistani data set for the first time.

## Results

### Socioeconomic characteristics of the participants

Table 2 shows the percent distributions of the 6977 women who delivered either at home or at a health facility based on their background characteristics. Women with a secondary education were more likely to deliver at a health facility (73%) than those who had no education (36%); similarly, women whose husbands were highly educated (57%) mostly preferred to deliver at a health facility, whereas those whose husbands were not educated did not (34%). As expected, among the high income quantiles, 73% of mothers visited health centres to deliver their babies, whereas this ratio was substantially lower among mothers belonging to the lowest income quantile (34%).

**Table 2:** Percent distribution of women who are having deliveries at home by their background characteristics

Characteristics	Place of birth %		*P*-value	N (6977)
	Health Facility	Home		
Education of Woman			0.000	
No education	36.03	63.97		4041
Primary	57.19	42.81		974
Secondary	73.20	26.80		1239
Higher	51.00	49.00		723
Education of Husband			0.000	
No education	34.00	66.00		2269
Primary	45.61	54.39		969
Secondary	57.16	42.84		2262
Higher				
Economic Status			0.000	
Poor	33.62	66.38		3067
Middle	48.70	51.30		1386
Rich	73.38	26.62		2524
Woman Occupation			0.000	
Not working	53.06	46.94		5537
Agriculture	27.11	72.89		461
Services	54.17	45.83		624
Others	44.07	55.93		355
Region			0.000	
Punjab	55.28	44.72		2006
Sindh	65.62	34.38		1585
KPK	47.49	52.51		1531
Baluchistan	25.83	74.17		1146
GB	54.44	45.56		709
Residence			0.000	
Urban	64.93	35.07		2971
Rural	40.66	59.34		4006
Age			0.000	
15–24	55.24	44.76		1633
25–34	51.98	48.02		3678
35–49	44.66	55.34		1666
Decision of woman’s medical treatment			0.000	
By Self	57.11	42.89		3033
Others	46.45	53.55		3944
Transport Availability			0.000	
Problem	40.12	59.84		3459
Not a Problem	61.65	38.35		3518
Travelling Alone			0.000	
Problem	44.37	55.63		4215
Not a Problem	61.12	38.88		2762

Women working in the agriculture sector had the lowest percentage (27%) of those who chose to deliver their babies at a health facility, whereas the women who served in the service sector had the largest proportion (54%). Across the five geographical regions (provinces), Sindh had the highest percentage (66%) of women who preferred giving birth at locations other than home, followed by Punjab (55%), Gilgit-Baltistan (54%), Khyber-Pakhtunkhwa.

(47%) and Baluchistan (26%).The percentage of women who delivered at a health facility was substantially higher in urban areas (65%) than in rural areas (41%). Mothers in the 15–24-year-old age bracket had the highest percentage (55%) of those who decided to deliver babies at health facilities instead of at home, followed by the 25–34 (52%) and 35–49 (45%) year old age groups. The decision about whether to visit a health centre at the time of birth was often made by the women themselves (57%), whereas in 46% of cases other members of the family made this decision. Mothers facing difficulty in transportation mostly deliver at home (60%) whereas mothers with no transportation issue are less likely to deliver at home (38%). Mothers who can travel alone have higher percentage (61%) to use health facility for delivery as compared to mothers who cannot travel alone (44%).

### Decision making concerning the birth location

#### Bivariate analysis

The bivariate analysis showed that decision making about delivering a baby at home or at a facility was the outcome of multiple factors that included permission from family members, money, distance to the health facility, transportation and the need to travel alone to the health facility. Table 3 reveals the percent distributions of women who delivered at home during the 5 years preceding the survey. Table 3 obviously showed that obtaining permission to deliver at a heath facility became easier as the education level of the mother increased. Of the women with no education, 39% reported that obtaining permission was a problem, whereas only 12% of women with higher education considered obtaining permission a problem.

**Table 3:** Percent distribution of women, 5 years preceding the survey, given birth at home

Characteristic	Reason for delivering at home										N
	Getting a permission to go is		Money is		Distance to health facility is		Transport is		Going alone is		
	Problem	No problem	Problem	No problem	Problem	No problem	Problem	No problem	Problem	No problem	
Education of Woman	X^2^ = 79.5951		X^2^ = 135.5945		X^2^ = 143.7967		X^2^ = 156.0720		X^2^ = 98.2706		
No education	38.99	61.01	55.74	44.26	61.55	38.45	66.27	33.73	72.84	27.16	2585
Primary	25.36	74.64	37.80	62.20	40.91	59.09	66.27	33.73	56.70	43.30	418
Secondary	21.62	78.38	30.93	69.07	38.44	61.56	42.34	57.66	57.36	42.64	333
Higher	12.94	87.06	21.18	78.82	25.88	74.12	28.24	71.76	41.18	58.82	85
Economic Status	X^2^ = 75.0153		X^2^ = 241.1833		X^2^ = 268.0461		X^2^ = 367.2921		X^2^ = 169.7331		
Poor	40.64	59.36	60.79	39.21	66.83	33.17	73.27	26.73	76.81	23.19	2035
Middle	29.35	70.65	40.59	59.41	46.21	53.79	48.17	51.83	60.67	39.33	712
Rich	23.89	76.11	28.78	71.22	33.09	66.91	35.01	64.99	52.08	47.92	674
Region	X^2^ = 527.8957		X^2^ = 444.4570		X^2^ = 463.6556		X^2^ = 522.0422		X^2^ = 272.4123		
Punjab	11.59	88.41	23.08	76.92	26.53	73.47	29.32	70.68	47.27	52.73	897
Sindh	28.07	71.93	44.59	55.41	60.73	39.27	66.97	33.03	70.64	29.36	545
KPK	40.92	59.08	65.30	34.70	66.04	33.96	66.79	33.21	80.35	19.65	804
Baluchistan	62.09	37.91	67.25	32.75	73.83	26.17	77.11	22.89	77.00	23.00	852
GB	25.39	74.61	53.25	46.75	56.66	43.34	76.78	23.22	72.76	27.24	323
Residence	X^2^ = 16.9964		X^2^ = 74.7497		X^2^ = 152.1964		X^2^ = 131.7937		X^2^ = 85.0209		
Urban	29.91	70.09	39.12	60.88	40.08	59.92	46.02	53.98	57.53	42.47	1043
Rural	37.22	62.78	55.17	44.83	55.89	44.11	66.86	33.14	73.42	26.58	2378

Obtaining permission was a big problem for poor women (41%) compared to rich women (24%). A substantial percentage of women living in rural areas (38%) considered permission a big issue, whereas 30% of women living in urban areas reported that obtaining permission was an issue. Baluchistan had the highest percentage (62%) of mothers who reported that permission was an obstacle, whereas this percentage was low (12%) in Punjab. Financial constraint was a critical factor that affected the decision to deliver the baby at home or at a health facility.

The percentage of women who reported money as a problem was highest among the women who were uneducated (56%), poor (61%), living in rural areas (55%) or from Baluchistan (67%); conversely, this percentage was lowest among the mothers who were educated (21%), rich (29%) living in urban areas (39%) and from Punjab (23%). Table 3 shows that the distance to a facility, transportation and the need to travel alone to the facility, were other obstacles that restricted women from delivering at a health facility. However, the graveness of these hurdles softened with increases in education, improvement in the economic status, residence in urban areas and a background from the Punjab province. For example, women living in urban areas considered distance from the facility (40%), transportation (46%) and traveling alone (58%) to be problems, but these percentages were higher for women who lived in rural areas (56, 67 and 73%, respectively).

A similar trend was witnessed for rich women compared with their poor counterparts. Women living in Punjab province were the least concerned about the distance to a health facility (26%), transportation (29%) and traveling alone (47%), whereas mothers living in Baluchistan were substantially more concerned about these factors (74, 77 and 77%, respectively). The percentages of rich mothers who mentioned the distance to a health facility, transportation and traveling alone to the facility centre were 33, 35, and 52%, respectively, compared to women belonging to the lowest income quintile (67, 74, and 77%, respectively). All of the relationships between the independent and dependent variables were significant (*p* < 0.01).

### Multivariate analysis

Table 4 reports the odds ratios for women who had deliveries at home instead of at any health facility in the 5 years preceding the survey. In model 1, we show that female autonomy measured by the woman’s education, the husband’s education and the decision of the woman about medical treatment are factors that determine the mother’s decision to deliver at home. The education of the mother is strongly linked with the decision of birth location, and mothers with higher levels (primary to high) of education are less likely to deliver their babies at home than those who have no education (primary, odds ratio (OR) 0.49; *p* = 0.000; secondary, OR 0.26; *p* = 0.000; and high, OR 0.11; *p* = 0.000). The husband’s education level is a strong predictor of the decision of birth location, and women with educated husbands are less likely to deliver at home than women with illiterate husbands. The probability of delivering at home decreased sharply with the improvement in the husband’s education level (primary, OR 0.73; *p* = 0.000; secondary, OR 0.62; *p* = 0.000; and high OR 0.52; *p* = 0.000).

**Table 4:** Odds Ratio of women, 5 years preceding the survey, giving births at home instead of any health facility

Indicators	Model-1	Model-2	Model-3	Model-4
	LR Test: *P*-value = 0.000	LR Test: *P*-value =0.000	LR Test: *P*-value = 0.000	LR Test: *P*-value = 0.000
Education of Woman (No education)^R^				
Primary	0.492^***^ (0.425,0.571)	0.503^***^ (0.434,0.583)	0.513^***^ (0.442, 0.595)	0.686^***^ (0.585,0.805)
Secondary	0.261^***^ (0.224,0.304)	0.270^***^ (0.231,0.314)	0.277^***^ (0.273, 0.323)	0.445^***^ (0.375, 0.527)
Higher	0.108^***^(0.083,0.139)	0.109^***^ (0.084,0.141)	0.110^***^ (0.085, 0.142)	0.206^***^(0.157,0.271)
Education of Husband (No education)^R^				
Primary	0.726^***^ (0.619,0.852)	0.734^***^ (0.625,0.861)	0.743^***^ (0.633,0..873)	0.855^*^(0.722,1.013)
Secondary	0.616^***^ (0.540, 0.702)	0.633^***^ (0.554,0.723)	0.635^***^ (0.556,0.725)	0.694^***^ (0.602, 0.799)
Higher	0.521^***^(0.442,0.616)	0.542^***^(0.458,0.640)	0.538^***^ (0.455,0.636)	0.644^***^ (0.537,0.773)
Decision of woman’s medical treatment (Others)^R^				
Self	0.834^***^ (0.750,0.926)	0.824^***^ (0.741,0.917)	0.809^***^ (0.726,0.900)	0.812^***^ (0.731, 0.919)
Woman Occupation (No work)^R^				
Agriculture		1.656^***^ 1.325,2.069)	1.648^***^ (1.319,2.060)	1.556^***^ (1.224,1.979)
Sales		1.177^*^ (0.976,1.421)	1.157 (0.958,1.397)	1.256^**^ (1.030,1.532)
Others		0.977(0.776,1.230)	0.975 (0.774,1.228)	1.167 (0.913, 1.491)
Age (15–24)^R^				
25–34			1.777^**^ (1.035,1.339)	1.249^***^ (1.091,1.4302)
35–49			1.236^***^ (1.061,1.440)	1.353^***^ (1.152,1.588)
Economic Status (Poor)^R^				
Middle				0.723^***^ (0.620,0.843)
Rich				0.441^***^ (0.371, 0.525)
Region (Punjab)^R^				
Sindh				0.456^***^(0.386, 0.538)
KPK				1.103 (0.938,1.297)
Baluchistan				2.565^***^(2.118,3.107)
GB				0.761^**^ (0.613,0.943)
Residence (Urban)^R^				
Rural				1.323^***^ (1.156,1.513)
Transport Availability (Problem)^R^				
				0.852^**^(0.738,0.984)
Travelling Alone (Problem)^R^				
				0.826^***^(0.732,1.037)
Constant	2.432^***^	2.274^***^	1.983^***^	1.947^***^
Hosmer-Lemeshow test (*P*-value)	0.513	0.172	0.259	0.319

*10% level of significance;**5% level of significance; ***1% level of significance

() Confidence Interval at 95% level of significance

Women who independently made decisions about their medical treatment were less likely to give birth at home (OR 0.83; *p* ≤ 0.001) than women who depended on other members of the family for decision making about health treatment. Model 2 controls for the occupations of the women. Women working in the agriculture and sales sectors were more likely to deliver at home (OR 1.65; *p* = 0.000 and OR 1.17 *p* = 0.000, respectively) than women who did not work. However, women engaged in other sectors of the economy showed a non-significant trend towards a reduced probability of delivering at home (OR 0.98; *p* ≥ 0.10) compared to non-working women.

In the third model (model 3 of Table 4), the woman’s age is included as control variable. It is evident that with the increase in age a mother is more likely to deliver at home as compared to young mothers (OR 1.77; *p* ≤ 0.05 for age group 25–34 years and OR 1.23; *p* = 0.000 for age group 35–49 years). This finding is logical as young mothers are more educated and more aware; therefore they prefer to deliver at health facility instead of home. Older, higher parity mothers are less likely to use health facility than younger, lower parity mothers [35, 36]. However, previous empirical literature shows varying results for the age groups of mothers in the context of decision to deliver at home or health facility. For example mothers having previous successful births at home with no complications are more likely to give birth at home [6], but on the other hand with the increase in age mothers may assume more role in decision making and, therefore, more likely to give birth at health. Previous studies also show curvilinear relationship between age and the use of skilled attendants at childbirth [37, 38].

The final model (model 4, Table 4) includes some background characteristics, such as the mother’s economic status, region, place of residence, access to transportation and decision to travel alone because these factors can affect the outcome, (i.e., the woman’s decision about childbirth). All five variables showed significant associations. The probability of delivering at home was lower for middle income (OR 0.72; *p* = 0.000) and rich (OR 0.44; *p* = 0.000) women than for poor women. The regional background and the mother’s place of residence were strong predictors of the decision to deliver at home. As expected, women living in Baluchistan and Khyber-Pakhtunkhwa were more likely (OR 2.56; *p* = 0.000 and OR 1.10; *p* ≤ 0.10, respectively) to deliver at home, whereas women from Sindh and Gilgit Baltistan were less likely (OR 0.45; *p* = 0.000 and OR 0.76; *p* ≤ 0.05, respectively) to deliver their babies at home. Mothers who lived in rural areas had a higher probability (OR 1.32; *p* = 0.000) of choosing home birth than women living in urban areas. Women who can avail transportation are less likely to deliver at home (OR 0.85; ≤0.05) and mothers that feel no issue in travelling alone are less likely to give birth at home (OR 0.82; *p* = 0.000).

**Fig. 1:**
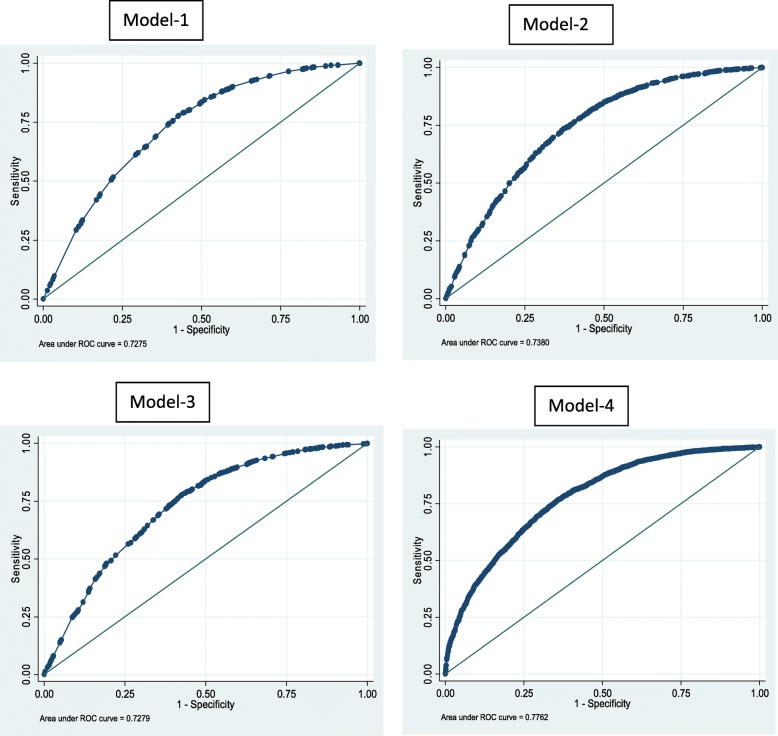
ROC curve test. Receiver operating characteristic (ROC) curve test shows prediction powers of the estimated models and its value should be greater than 0.7. All the estimated models i.e. Model 1, Model 2, Model 3, Model 4 have the area under the ROC curve fairly greater than 0.7

After controlling for all odds, we noted that the education levels of the mothers and their husbands and the autonomy of the women remained significant predictors of the decision of birth location. Lastly, we performed post-estimation validation of our results using a ROC curve test. The Fig. 1 shows that all of the estimated models have fair prediction powers (i.e., the value of the area under the curve is greater than 0.7).

## Discussion

Clear decision making in pregnancy and birth is challenging for women and childbirth stress and hospital context could complicate decision of mothers during labor and delivery [13]. Similar to the situation in many developing countries, the majority of women in Pakistan still prefer to deliver at home over giving birth in a health facility [7]. This preference could be due to unplanned deliveries and varying socio-demographic statuses [39]. This study is important because it explores the reasons behind women’s decisions to deliver babies at home using PDHS 2012–13, which is the latest available data set in Pakistan. Our study established that obtaining permission to visit a health facility, financial constraints, distance to the facility, transport and traveling alone to a health facility were the major factors that shaped a woman’s decision to deliver at home. However, the percentages of women mentioning the above barriers to delivering a baby at a health facility substantially varied when their background socioeconomic and demographic characteristics were considered. Previous studies conducted in different parts of the world have explored factors associated with the place of birth and obtained results that are in line with our findings [7].

This study revealed that the education level of the mother and husband remains significant factors that affected women’s decisions to deliver at home rather than at a health facility after controlling for all known factors and in all models. Previous empirical studies conducted in different regions of the world reported that the educational status impacted women’s decisions to seek care from hospitals [3, 40–42]. Education enhances mother’s abilities to afford health facilities, and concerns about health, autonomy, and freedom to make decisions impact their decisions to deliver at home or at a health facility [30].

The findings show that the mother’s ability to make decisions about medical treatment lessens the probability of delivering babies at home in all settings (*p* = 0.000). Limited access of women to decision making is one reason for delivering a baby at home in Bangladesh [31]. Joint decision-making with families, household dynamics and perceived quality of care are also influential in determining mother’s decisions to seek care [16]. For instance, Laotian women reported that their comfort levels increased if their husbands and other family members were nearby to provide support [43].

This study concludes that home birth decisions heavily depend upon women’s occupations. As expected, women engaged in the agriculture and services sectors were more likely to not prefer birth at a health facility than women working at home. The probability of mothers working in the agriculture sector delivering at home was 1.5 times higher than the probability of delivering at a health facility [7]. The woman’s age at the time of birth was a strong predictor of decision making about whether to deliver at home or at a health centre, with young women more likely (*p* ≤ 0.05) to prefer to deliver babies at health facility. This finding is contrary to previous studies who found that young women, notably those between the ages of 15 and 24 years, were more likely to deliver at home than older mothers [44].

Our study highlights that women who belong to poor families are 2 times more likely (*p* = 0.000) to undergo home confinement than women who hail from rich families. This result is in agreement with the findings of many other studies. For example, a study conducted in Pakistan showed that the postnatal care (PNC) utilization rate was higher in the rich stratum than in the poor stratum [45]. Likewise, another study in the context of Pakistan showed that poor families could not afford the high cost of birth at a private clinic or even at a government hospital, which left women with no option but to deliver at home [31]. Deliveries at health centres require patients to pay for costs, such as medicines and transport, which poor people cannot afford [46, 47]. A study conducted in rural Burkina Faso revealed that money was a major barrier to using a health facility as a place of birth and that poor women delivered at home due to this financial constraint with the expectation that all would go well [48]. A study in Ethiopia established that women with higher income are more likely to avail institutional facility to deliver baby [49].

Substantial variation was observed across the five provinces. Women from the least developed province (Baluchistan) were more likely (OR 2.56; *p* = 0.000) to deliver at home than women from the well-developed Punjab province. Interestingly, women in the newly created Gilgit Baltistan province were less likely to deliver at home than women from Khyber-Pakhtunkhwa and Baluchistan. Research conducted in Pakistan revealed that PNC utilization by mothers was lowest in Baluchistan [50].

The findings show that a woman’s place of residence (i.e., urban or rural) is a key factor that determines women’s decisions about their delivery locations. Women living in rural areas are 1.32 times more likely to deliver at home than their counterparts living in cities. The long distance to health centres may be an explanation for this result. Similar findings were reported in a study conducted in Tanzania, which stated that long distances to health facilities in rural areas were a critical determinant of home birth [18]. The findings obtained in this study are similar to correlates of home birth across multiple nations.

This study established that transport service availability is strong predictor of women’s decision to deliver at home or health facility particularly in rural areas as women could develop labor at night when public transport is not available. This finding is fully supported by studies done in other countries. The pregnant women in rural Africa has limited access to transport services when they develop labor because of deteriorating condition of road nets and infrastructure [51, 52]. Main barrier, among others, to use health facility among Indian women is cost of arranging transportation to the facilities [16]. In Tanzania 84% of women having delivered at home intend to deliver at health facility instead of home but could not due to transportation related issues [53].

Decision to travel alone, which is one of the component of women empowerment, encourages women to deliver at health facility. Other studies conducted in Pakistan also show the negative impact of women empowerment on home births [7, 54]. Yet in developing countries like Pakistan, travelling alone for women is uncommon and is considered a stigma in least rural areas where tribal and feudal systems are prevalent.

In brief, decision making in pregnancy and birth is challenging and complex [13]. The wholesale shift to hospital birth may be a slow process that requires some community based measures to decrease maternal mortality. Sri Lanka has developed its primary health care system by providing skills to primary health workers in rural areas and retaining them in remote regions of the country. Pakistan too can follow this model to overcome the high prevalence of pregnancy and birth related mortalities particularly in its least developed areas. Moindi et al. [6] also suggest more investment in rural areas health facilities. This can be achieved by launching programs in remoted areas to reduce skills gaps and ensuring the availability of qualified healthcare professionals in poor areas of the country.

The above findings have also some gender dimensions. For example, according to World Economic Forum, Pakistan has been ranked as the second worst country in the world in the gender inequality index [55]. In Baluchistan province women are oppressed due to male domination [22], face injustice because of misinterpretation of Islamic values [56], remain illiterate as parents expect no returns in investing their education [21], their birth is considered burden and are forced to observe purdah [23], denied share in heritage and have no role in decision making.

Similar to many other studies on the subject, this study is not free from limitations. Among other limitations, more information about the group of women categorized as not working is not available which may include women from higher economic strata who do not need to work as well as those unable to find work. These groups of women would be expected to have differing rates of birth in health facilities versus home births. Further, robust conclusions cannot be inferred from our findings because the data used for the analysis are cross sectional in nature.

## Conclusion

This study explored the factors that influenced women’s decisions to give birth at home or at a health facility in Pakistan using the most recently collected data from the PDHS. A wide range of factors, including financial barriers, female autonomy, distance to a health facility, transportation and the need to travel alone to the health centre, emerged as strong predictors of the decision of birth location when observed in the context of the socioeconomic and demographic background. Substantial variation was found in the results due to the educational levels of the women and their husbands, the economic status of the mothers, the provinces to which they belonged, and their places of residence, ages, transport service facility and their decision to travel alone. The findings from this study may help policymakers and implementers frame efficient strategies to encourage women to deliver at health facilities instead of at home. For example, the launch of educational and awareness campaigns aimed at enhancing women’s understanding and knowledge about maternal delivery care could be effective. The need to ensure the availability, accessibility, and affordability of health facilities to mothers irrespective of their background characteristics cannot be over emphasised. For instance, the provision of quality health facilities in remote and hard-to-reach areas may be a good option to reduce prevalent home birth trends. Removal of transportation barriers by compensating women for transport costs can have an impact on lowering the proportion of home deliveries. The husband’s involvement in the decision to choose a health facility for birth of the baby can be increased by educating husbands through various interventions. Trends toward an early age at marriage, especially in rural areas, need to be discouraged to reduce maternal deaths during deliveries at home by launching community awareness programs because young mothers are more likely to deliver at home than older women. Baluchistan province requires special attention because persistent evidence [7, 50] shows that women in this province prefer home confinement due to strong cultural norms. To overcome the problem of transport services especially in rural areas, the introduction of “waiting homes” near the health facilities to accommodate expectant mothers belonging to remote areas could be effective [57]. By educating rural women to become skilled birth attendants, number of deaths could be averted during pregnancy. This intervention has been quite successful in Sri Lanka.

A caution is in order, however, that above-mentioned strategies could only be effective if an integrated policy aimed at winning the trust of all partners and participants in perinatal care delivery system is designed as trust, at least to some minimal extent, is undoubtedly a prerequisite to seeking care at all [58–60]. It must be remembered that behavioural change occurs gradually and to make an impact on the ground government must know the dynamics and feel constraints in the health system [61].

## Abbreviations

ANC: Antenatal services
GB: Gilgit Baltistan
MDG: Millennium development goal
MMR: Mother mortality ratio
OR: Odd ratio
PDHS: Pakistan demographic and health survey
ROC: Receiver operating characteristic
